# Tracking Monochloramine Decomposition in MIMS Analysis

**DOI:** 10.3390/s20010247

**Published:** 2019-12-31

**Authors:** Adrien Roumiguières, Said Kinani, Stéphane Bouchonnet

**Affiliations:** 1 Laboratoire National d’Hydraulique et Environnement (LNHE), Division Recherche et Développement, Electricité de France (EDF), 6 Quai Watier, 78401 Chatou, CEDEX 01, France; adrien.roumiguieres@polytechnique.edu (A.R.); said.kinani@edf.fr (S.K.); 2 Laboratoire de Chimie Moléculaire, CNRS, Institut Polytechnique de Paris, Route de Saclay, 91128 Palaiseau, France

**Keywords:** monochloramine, membrane-introduction mass spectrometry, Fourier transform-ion cyclotron resonance, adsorption, surface-catalyzed decomposition

## Abstract

Membrane-introduction mass spectrometry (MIMS) has been presented as one of the promising approaches for online and real-time analysis of monochloramine (NH_2_Cl) in diverse matrices such as air, human breath, and aqueous matrices. Selective pervaporation of NH_2_Cl through the introduction membrane overcomes the need for sample preparation steps. However, both the selectivity and sensitivity of MIMS can be affected by isobaric interferences, as reported by several researchers. High-resolution mass spectrometry helps to overcome those interferences. Recent miniaturization of Fourier transform—ion cyclotron resonance—mass spectrometry (FT-ICR MS) technology coupled to the membrane-introduction system provides a potent tool for in field analysis of monochloramine in environmental matrices. Monochloramine analysis by MIMS based FT-ICR MS system demonstrated decomposition into ammonia. To further clarify the origin of this decomposition, headspace analyses after bypassing the membrane were undertaken and showed that monochloramine decomposition was not exclusively related to interactions within the membrane. Adsorption inside the MIMS device, followed by surface-catalyzed decomposition, was suggested as a plausible additional mechanism of monochloramine decomposition to ammonia.

## 1. Introduction

Monochloramine (NH_2_Cl) has useful applications in many important industrial activities. It is widely used as a disinfection and/or antifouling agent [1,2,3]. NH_2_Cl may also be formed as a constituent of “chlorine-produced oxidants” during chlorination of water with high ammonium content, such as coastal waters [4]. Online analysis of NH_2_Cl is of major interest because it addresses various questions related to treatment efficacy and environmental impact. For example, determining NH_2_Cl concentrations in seawater contributes to the chemical speciation of chlorine-derived oxidants, which is helpful in understanding chlorine chemistry and to optimize treatment. In seawater ammonia competes with bromide to react with chlorine. Several studies reported that formation of NH_2_Cl dominates that of bromine (Br_2_/HOBr/^−^OBr) in chlorinated seawater when ammonia concentration exceeds 0.4 ppm [5,6]. At ammonia concentrations over 0.1 ppm, NH_2_Cl concentration is higher than bromamines concentration [7]. Compared to bromamines, which are very unstable and decompose rapidly, NH_2_Cl can remain longer in chlorinated seawater [8]. Being a weaker oxidant than free bromine, monochloramine has a longer half-life and produces significantly less total organic halogen compounds such as trihalomethanes and haloacetic acid.

However, monochloramine analysis at low concentrations presents significant analytical challenges due to its physicochemical properties: Unstable in water, relatively low molecular weight, high polarity, good water solubility, and absence of chemical groups that might facilitate detection. Much effort has been devoted in recent years to the development of sensitive and robust methods to measure NH_2_Cl concentrations in different aqueous matrices [1,2]. Several previous studies presented membrane-introduction mass spectrometry (MIMS) as one of the promising approaches for online and real-time analysis of monochloramine [1,9,10]. In this technique, analytes of interest are introduced into a mass spectrometer through a membrane, according to the principle of pervaporation. Polydimethylsiloxane (PDMS) is the most common used permselective membrane material. Mass spectrometry enables analytes to be identified by their mass-to-charge ratio. The amplitude of the product ion signal gives a quantitative measurement of analyte concentrations in the sample. The feasibility of MIMS for online screening of inorganic haloamines in water samples was examined in several studies [11,12,13,14,15]. Literature backgrounds regarding inorganic chloramines analysis using the MIMS method are summarized in Table S1 (given in Supporting Information). Current applications for NH_2_Cl analysis mainly rely on electron ionization (EI) at 70 eV on low-resolution mass analyzers (usually quadrupoles, or sometimes ion traps). The resulting fingerprints comprise complex spectra of many overlapping fragments, which does not allow isobaric compounds to be distinguished. Detection limits were often too high to be useful [1].

Recently, we investigated a novel transportable MIMS system equipped with a compact Fourier transform—ion cyclotron resonance mass spectrometer (FT-ICR MS) based on a permanent magnet (1.5 Tesla), allowing high resolution in the range 15–300 amu. One of the advantages of this instrument is its ability to discriminate between ions of interest and isobaric interference, in contrast to commonly used low-resolution analyzers: A simple quadrupole for Li and Blatchley [9], triple quadrupole for Yang and Shang [16], and ion trap for Weaver et al. [17]. In addition, this instrument uses hydronium ions (H_3_O^+^) as a chemical ionization (CI) reagent. The acquired mass spectra are simpler, because proton transfer reaction (PTR) CI is “soft” and causes little or no fragmentation, resulting in higher sensitivity. PTR ionization of NH_2_Cl can be represented by the following reaction:

NH_2_Cl + H_3_O^+^ → NH_3_Cl^+^ + H_2_O (1)

According to this ionization reaction, protonated NH_2_Cl should present at two peaks, *m*/*z* 51.995 (NH_3_^35^Cl^+^) and 53.992 (NH_3_^37^Cl^+^), due to the isotopic distribution of the chlorine atom. However, contrary to our expectations, the acquired mass spectrum also had an intense ion at *m*/*z* 18.034, which is attributed to the NH_4_^+^. The recent study by Louarn et al. suggests that this ion results from monochloramine decomposition inside the membrane [11]. This decomposition has never been reported in other studies. In the present study, an aqueous NH_2_Cl solution was analyzed on an FT-ICR device of the same type, with and without membrane introduction (MIMS versus headspace-MS) to accurately determine where NH_2_Cl decomposition occurred and whether it could be avoided.

## 2. Materials and Methods

### 2.1. Reagents and Chemicals

All reagents and chemicals were of analytic quality. Ammonium chloride (purity > 99.5%), sodium hypochlorite (13% as Cl_2_), and sulfuric acid (purity > 95.0%) were purchased from Sigma-Aldrich (Steinheim, Germany); sodium hydroxide (purity > 99.0%), from Merck (Darmstadt, Germany); DPD (N,N-diethyl-p-phenylenediamine) kits for “free and total chlorine” analysis, from Hach (Lognes, France); and aqueous solutions were prepared with ultrapure water (specific resistance, 18 MΩ cm^−1^ at 25 °C) produced by a PURELAB Chorus 1 water purification system purchased from Veolia Water Technologies (Wissous, France).

### 2.2. Monochloramine Preparation and Standardization

Monochloramine is unstable in aqueous media and no standards are commercially available. NH_2_Cl solutions were prepared fresh daily, as described by Kinani et al. [2,3]. To reduce ammonia traces in standard solution, an excess of chlorine was added to ammonium chloride (molar Cl:N ratio = 1.05:1). The pH of the NH_2_Cl solution was then adjusted between 7 and 7.2 by dropwise addition of 1 N NaOH and H_2_SO_4_. At this pH value, 99% of residual ammonia traces are in the NH_4_^+^ form (pKa (NH_4_^+^/NH_3_) = 9.25), which is not volatile. NH_3_ and NH_2_Cl have similar volatilities, with Henry’s law constants of 0.59 and 0.86 mol m^−3^ Pa^−1^, respectively [18]. Under those experimental conditions, NH_3_ can be considered negligible compared to NH_2_Cl. The resulting monochloramine solutions were standardized by DPD colorimetry, following the procedure described in the Standard Method NF EN ISO 7393-2 [19].

### 2.3. Instrumentation and MIMS Analytical Conditions

“Free and total chlorine” were measured using DPD method-based test kits (Hach #1406428, Loveland, CO, USA) and a DR 2800 UV-visible spectrophotometer with 2.5 cm quartz cell (Hach, Loveland, CO, USA). During chlorination of ammonium chloride at different Cl:N ratios, a double-beam Jenway 6800 UV-visible spectrophotometer with 5 cm quartz cells was used (Jenway, Essex, UK). For all samples, spectra were recorded in the 190–500 nm range against blank, using sampling intervals of 0.2 nm with a scan speed of 500 nm min^−1^. pH was measured with a “SevenEasy” pH-meter from Mettler Toledo (Columbus, OH, USA).

MIMS experiments were performed using a 1.5 Tesla BTrap™ Fourier transform-ion cyclotron resonance-mass spectrometer (Alyxan, Juvisy-sur-Orge, France) equipped with an internal ion source. A similar system has been described in detail elsewhere [20,21]. Analysis was performed by chemical ionization using H_2_O as reagent. The proton affinity (PA) of monochloramine (PA = 797.05 kJ·mol^−1^) exceeds that of H_2_O (PA = 691.0 kJ mol^−1^), signifying that monochloramine might be able to undergo proton transfer reaction with H_3_O^+^, as shown in Equation (1) [22,23]. Hydronium ions were generated inside the ICR cell from water vapor pulses after electron ionization and molecular ion reactions. The FT-ICR MS acquisition required optimization of a set of parameters: Water as ionization reagent; trimethylbenzene as internal standard for mass calibration, and sample introduction times; reaction time; trap and exciting potentials. The optimized analytical sequence presented in Figure S1 (given in Supporting Information) was adapted from Louarn et al. [20]. The FT-ICR MS acquisition sequence for all experiments was as follows: After the quench event (to remove ions from the ICR cell), water from the reservoir was pulsed into the ICR cell for 15 ms (*t* = 0 to 15 ms). After a short interval of 35 ms (*t* = 15 to 50 ms), the water reagent was ionized by 70 eV electrons for 25 ms (*t* = 50 to 75 ms). A reaction time of 415 ms was used to generate H_3_O^+^ ions. These reagent ions were isolated between *t* = 490 to 491 ms using stored waveform inverse Fourier transform (SWIFT), prior to introducing the sample vapor and trimethylbenzene via a separate pulsed valve for 500 and 5 ms, respectively. After an interval of 1260 ms (at *t* = 991 to 2251 ms) to allow the ICR cell region to return to near-baseline vacuum conditions, the ions were detected. The total cycle time was 5 s. To improve sensitivity, 10 spectra were summed, bringing the total cycle time to 50 s. The mass range scanned was *m*/*z* 15 to 150. The identities of the observed ions were confirmed by accurate mass measurement. Mass calibration was performed on H_3_O^+^ (19.0184 Da) and C_9_H_13_^+^ (121.1017 Da) ions. Mass errors for detected ions are given in the Supporting Information, Table S2. Over this mass range (18 to 100 Da), the high resolution of the FT-ICR MS device (<±0.003 Da) enabled reliable determination of targeted compounds. Prior to carrying out tests, the FT-ICR MS was kept isothermal at 150 °C for 24 h to release potentially adsorbed compounds on the walls. The vacuum chamber surrounding the ICR cell was set at 90 °C during the analysis phase.

**Figure 1:**
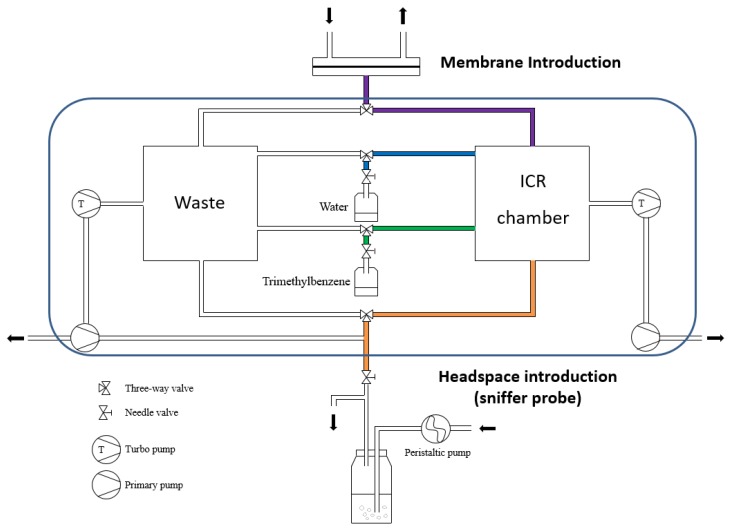
Diagram of the inlet system in the BTrap™ device.

The BTrap™ MS system is equipped by three possible sample inlets: (i) A membrane inlet for air, (ii) a membrane inlet for aqueous samples, and (iii) a sniffer probe for direct gas introduction. A schematic presentation of the instrument is given in Figure 1. A closed amber glass sample reservoir (1 L, septum equipped) was used for all experiments and was plumbed to the mass spectrometer. The headspace vapors distilled from aqueous solutions were delivered to the PTR-MS device through a PFA (perfluoroalkoxy) transfer line (Swagelok, PFA-T2-030-100, 30 cm length × 0.16 mm I.D) to minimize memory effects. A DOSE IT peristaltic pump from Integra Biosciences bubbled air into the water samples (flow rate, 100 mL min^−1^) to facilitate the volatilization and transfer of the analytes of interest into the MS system. A needle valve was positioned on the inlet line to adapt the headspace vapor flow rate to the pressure value required by the mass spectrometer, which should be around 10^−5^ mbar during sample introduction and 10^−8^ mbar at detection [21]. A three-way valve transferred the sampling flow intermittently and more accurately to the “Waste” before discarding or to the “ICR chamber” for analysis. The line—starting at the membrane position exit and extending to the entrance to the ion source—was maintained at 50 °C to minimize analyte adsorption. All experiments were carried out at room temperature (22 ± 1 °C). Before analyzing each sample, a blank spectrum was acquired.

## 3. Results and Discussion

### 3.1. Monochloramine Analysis in Water

The first tests were carried out using a membrane route (for liquid samples) in order to optimize mass spectrometric parameters and characterize the mass spectrum of monochloramine. Thus, parameters that affect membrane introduction, such as membrane apparatus temperature and sample flow rate, were firstly optimized for NH_2_Cl measurement. The membrane was a thin film of polydimethylsiloxane (PDMS) (Goodfellow, SI301126) with thickness of 125 µm and sampling area of 60 mm^2^. Each parameter was studied individually using NH_2_Cl aqueous solution at a concentration of 70 mg L^−1^ (as Cl_2_). The effect of sample flow rate was investigated at 1, 10, and 100 mL min^−1^ (at 22 °C membrane temperature). MIMS signals were examined at six temperatures: 20, 30, 40, 50, 60, 70, and 80 °C. Using a PDMS membrane similar to that used in this research, Louarn et al. reported that steady state was not reached after 15 min of analysis; the NH_2_Cl solutions were therefore circulated through the membrane for 45 min, to equilibrium response [11].

**Figure 2:**
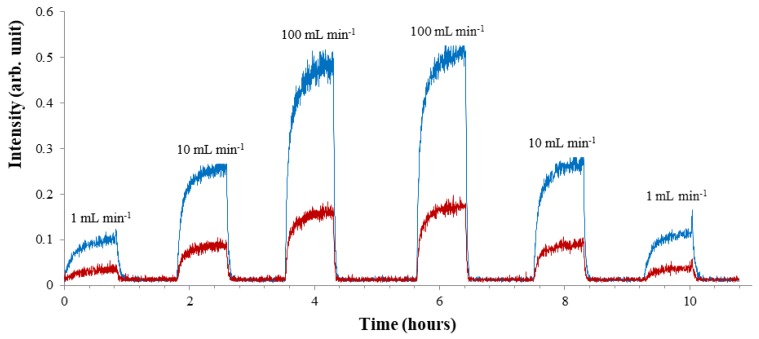
Influence of flow rate on Membrane Introduction Mass Spectrometry (MIMS) signal intensity. Ions with *m*/*z* values of 51.995 (NH_3_^35^Cl^+^: Blue) and 53.992 (NH_3_^37^Cl^+^: Red) were selected to monitor monochloramine. Experiments were conducted in duplicate.

Results (Figure 2) showed that signal intensity increased abruptly with increasing sample flow through the membrane (at room temperature (22 ± 1 °C)). This is consistent with results reported previously by She and Hwang [24]. By increasing the flow rate, turbulence along the membrane increases, the boundary layer decreases and analyte transfer is thus facilitated. The system reached maximum signal abundance at a sample flow rate of 100 mL min^−1^. Increasing the flow rate above this value could irreversibly damage the membrane and mass spectrometer. 100 mL min^−1^ was thus chosen as optimal flow rate and selected for membrane temperature studies. No significant effect of membrane temperature on MIMS signal intensity was observed at any values studied, and membrane temperature was therefore set at room temperature (22 ± 1°C).

**Figure 3:**
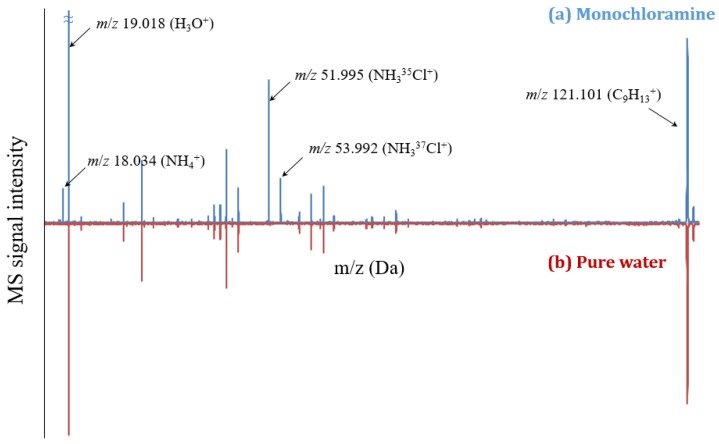
Mass spectrum obtained by MIMS for aqueous solution of monochloramine at a concentration of 70 mg L^−1^ (as Cl_2_) and pH between 7.0–7.2. The inversed spectrum at the bottom is from the ultrapure water (as blank).

Figure 3 shows the mass spectrum of the NH_2_Cl aqueous solution at a concentration of 10 mg L^−1^ (as Cl_2_). This spectrum agreed well with that reported by Louarn et al. [11]. The dominant peak at *m*/*z* 19.018 represents the H_3_O^+^ reagent ion signal, and the secondary peak at *m*/*z* 121.102 represents the C_6_H_3_(CH_3_)_3_H^+^ protonated trimethylbenzene. The characteristic peaks of protonated monochloramine were at *m*/*z* 51.995 (NH_3_^35^Cl^+^) and 53.992 (NH_3_^37^Cl^+^). Ricci and Rosi found that these ions showed the NH_3_-Cl^+^ structure [22,23]. In addition, the mass spectrum of NH_2_Cl solutions exhibited an intense peak at *m*/*z* 18.034, attributed to the NH_4_^+^ ions. Two hypotheses may be formulated to explain their formation: (i) Fragmentation of protonated monochloramine and/or (ii) protonation of ammonia present in the NH_2_Cl solution. The first hypothesis can be ruled out, since protonated monochloramine contains only three hydrogen atoms. An alternative route involving ion-molecule reactions, such as those given in Equations (2) and (3), could be suggested. However, the reaction time and hydronium ion pressure in the ICR cell make this mechanism unlikely.

NH_3_Cl^+^ + NH_2_Cl → NHCl_2_ + NH_4_^+^ (2)

NH_3_Cl^+^ + H_2_O → HOCl + NH_4_^+^ (3)

As described above (Section 2.2), the chlorine-to-nitrogen molar ratio was set at 1.05 to ensure complete transformation of ammonia into NH_2_Cl. If there is a free ammonia residual in the sample, it will be at more than 99% in the NH_4_^+^ form (pKa (NH_4_^+^/NH_3_) = 9.25), because the NH_2_Cl solution pH was adjusted 7.0–7.2. Note that MIMS yields mass spectral signals only for analytes that are able to pervaporate through the membrane. It is generally believed that hydrophobic membranes such as PDMS, used in this research, impede the diffusion of ionic and hydrophilic compounds [25]. This suggests that the detected NH_4_^+^ resulted from decomposition of monochloramine into ammonia inside the MIMS instrument. A similar conclusion was reported recently by Louarn et al., who suggested that this decomposition takes place across the membrane, since the low pressure inside the mass spectrometer source prevents adsorption and degradation reactions downstream of the membrane [11]. Monochloramine decomposition on membrane has never been reported in other studies. As discussed above, current applications for NH_2_Cl analysis mainly rely on electron ionization (EI) at 70 eV. If the decomposition of monochloramine had occurred in previous studies, a peak at *m*/*z* 17 (NH_3_^+^) and 18 (NH_4_^+^) should be observed in the reported spectra. These spectra are given in Supporting Information, Figure S2. However, the formation of these ions cannot be verified since the published mass spectra start at *m*/*z* > 35, to avoid the recording of water (H_2_O^+.^ at *m*/*z* 18, and H_3_O^+^ at *m*/*z* 19) and air (mainly: N_2_^+.^ at *m*/*z* 28 and, O_2_^+.^ at *m*/*z* 32) ions.

It should be noted that disproportionation of monochloramine into dichloramine (NHCl_2_) is possible at pH 7 [3,26]. However, no trace of characteristic NHCl_2_ ions was found, even though its proton affinity (PA = 757.0 ± 10 kJ mol^−1^) is higher than that of water [22,23]. This is likely due to the fact that the NHCl_2_ concentration in monochloramine solutions was below the MIMS detection limit. In order to accurately determine where NH_2_Cl decomposition occurs (and if it could be avoided), the sniffer route (without membrane) was used in the tests presented in the following sections.

### 3.2. Headspace Analysis of Ammonia (NH_3_/NH_4_^+^) and NH_2_Cl in Aqueous Solutions

**Figure 4:**
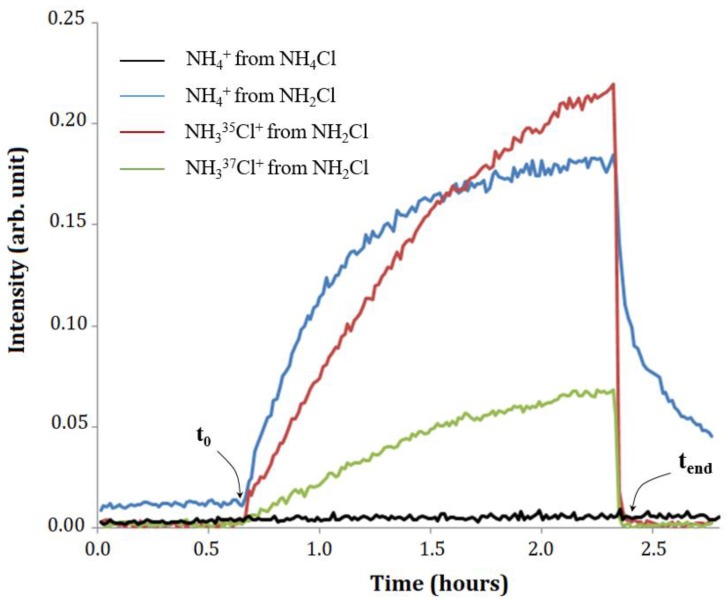
Kinetics of ion formation from a 20 mM NH_4_Cl solution (relative standard deviation (RSD) < 15%, *n* = 3). The two time-points, t_0_ and t_end_, represent the beginning of headspace introduction and time of solution removal, respectively.

In separate experiments, a 20 mM NH_4_Cl solution and a 20 mM NH_2_Cl solution were analyzed at neutral pH in headspace (without membrane) for 100 min. The NH_2_Cl synthesis protocol described in the Materials and Methods Section was used; it allows the ammonia concentration to be taken as negligible in comparison with the monochloramine concentration. The distilled vapors of the aqueous solutions were analyzed by FT-ICR MS according to the analytical sequence described above in Section 2.3. The headspace-MS profiles obtained are shown in Figure 4.

As illustrated in Figure 4, no signal of NH_4_^+^ ions (*m*/*z* = 18.034) was detected during headspace analysis of NH_4_Cl, presumably because of its low volatility at pH 7. In contrast, NH_4_^+^, NH_3_^35^Cl^+^, and NH_3_^37^Cl^+^ ions were detected as soon as the NH_2_Cl solution vapor was introduced. Their abundances increased as long as NH_2_Cl was being introduced, with a corresponding decrease in the abundance of reagent ions (H_3_O^+^). These results unambiguously confirm that NH_4_^+^ ions arise from NH_2_Cl decomposition, which does not occur exclusively inside the membrane. After 100 min of analysis, the NH_2_Cl solution was removed and replaced by ultrapure water. The intensities of NH_3_^35^Cl^+^ and NH_3_^37^Cl^+^ decreased rapidly in a few minutes, long enough to empty the entire line of introduction of NH_2_Cl vapor. In contrast, the abundance of NH_4_^+^ decreased much more slowly. After 30 min cleaning, there was still about a third of the maximum abundance observed during monochloramine analysis. This suggests that NH_4_^+^ ions do not arise from ion-molecule reactions involving NH_3_Cl^+^ and NH_2_Cl. Given the remanence of NH_4_^+^ ions, it is reasonable to assume that the ammonia adsorbs on the internal walls of the transfer line between the input of the analytical system and that of the ICR cell.

### 3.3. Headspace Analysis of Chlorinated Ammonium Chloride Solutions at Different Cl:N Molar Ratios

**Figure 5:**
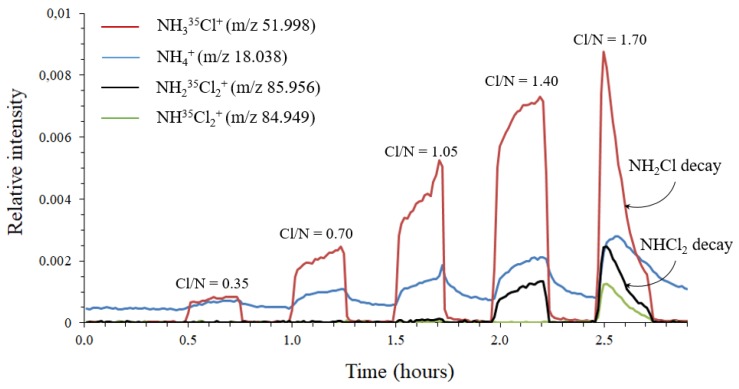
Stepwise chlorination of a 20 mM NH_4_Cl solution with different molar Cl:N ratios in headspace mode.

A 20 mM NH_4_Cl solution was chlorinated by successive additions of hypochlorous acid at molar Cl:N ratios of 0.35, 0.70, 1.05, 1.40, and 1.75. After each addition, pH was adjusted to 7.0–7.2 in order to minimize the ratio of NH_3_ to NH_4_^+^. Each mixture was analyzed in headspace for 15 min. Ultrapure water steam was introduced for 15 min to clean the system between each measurement. The chlorinated solutions were also monitored by UV-Vis spectrophotometry operating in the 200–500 nm wavelengths range. The spectra are given in Supporting Information, Figures S3 and S4. Figure 5 shows the evolution of ion abundance as a function of sample introduction time. For molar Cl:N ratios less than 1, ammonia is in excess over chlorine and only NH_3_Cl^+^ and NH_4_^+^ ions were detected by MIMS. Moreover, the concentration of monochloramine in solution increases while that of ammonia decreases with Cl/N (up to a ratio of 1). If NH_4_^+^ ions are produced by protonation of NH_3_ present in the aqueous solution, the intensity of NH_4_^+^ should decrease over time, which what was not observed in this study. This further confirms the conclusions of Section 3.2. At a Cl:N molar ratio of 1.40, the abundances of NH_4_^+^ and NH_3_Cl^+^ ions still increased. In addition, an ion appeared at *m*/*z* 85.956 (NH_2_Cl_2_^+^), indicating the formation of dichloramine, as also confirmed by UV-Vis spectrophotometry. Finally, increasing the Cl:N molar ratio to 1.75 led to a peak at *m*/*z* 84.949, corresponding to NHCl_2_^+^ ions. NHCl_2_^+^ is formed by protonation of trichloramine followed by the loss of a Cl atom [27]. The isotopic distributions of both NH_2_Cl_2_^+^ and NHCl_2_^+^ are confirmed in the Supporting Information, Figure S5. It is noteworthy that the abundance of all ions increased rapidly before decreasing suddenly. Monochloramine and dichloramine signal decrease may be explained in part by their conversion to trichloramine. Trichloramine is a volatile compound (Henry’s law constant of 9.9 × 10^−4^ mol m^−3^ Pa^−1^ [18]) and its concentration rapidly decreases, as confirmed by DPD measurements.

The transient state (due to pervaporation steps) should not be observed analyzing headspace vapor using a sniffer route. Ion intensities are expected to increase nearly instantaneously and then remain constant. However, the results showed that the MS signal continued to increase, as shown in Figure 5. This is consistent with the results reported by Hansen et al., with a second increase, slower than the first, explained by an adsorption phenomenon on the walls of the mass spectrometer between the introduction system and the ion source [28]. They also reported surface-catalyzed decomposition of phenoxyacetic acid and chloroethylenes into phenol and HCl/Cl_2_, respectively. To deal with this phenomenon, they advised minimizing the distance between the membrane and the ion source. They used a 2 cm long stainless-steel tube to connect the membrane inlet to the closed ion source. In the present research, the membrane inlet and ICR cell were spaced at more than 70 cm distance. Louarn et al. estimated that this phenomenon should not be observed, due to the low pressure inside the ICR cell [11]. The total pressure measured by Hansen et al. inside their ion source was 0.7 mTorr [28]. A 0.03 mTorr permeate pressure was measured inside the MI-FT-ICR MS used for their study. In the adsorption model presented by the authors, the amount of adsorbed molecules increased linearly with both wall surface area and pressure. In the present study, although the pressure was 20-fold lower inside the BTrap™ than in Hansen’s device, the internal surface area was at least 20-fold greater, so that adsorption should not be ignored. In both headspace and membrane introduction analysis, after removing the NH_2_Cl solution, the abundances of all ions decreased. The decrease was rapid for NH_3_Cl^+^ and reached baseline after less than 3 min. For NH_4_^+^, the decrease was much slower, and had not reached baseline after 30 min, a remanence of ammonia being observed.

### 3.4. Continuous Desorption of Adsorbed Ammonia

**Figure 6:**
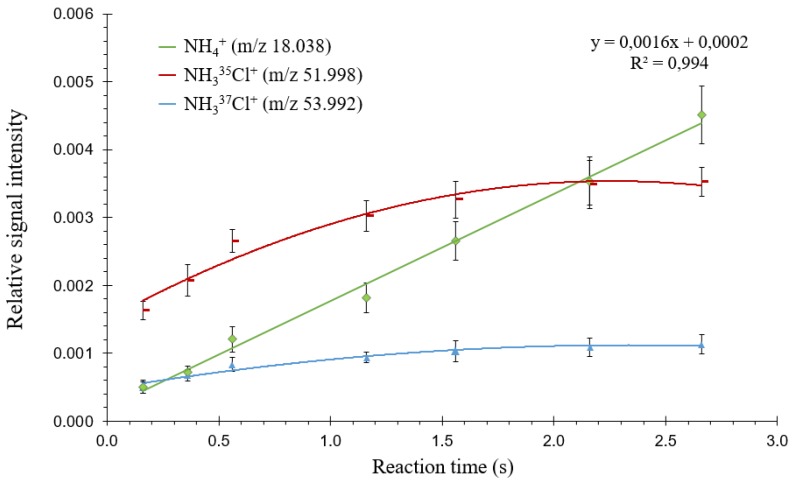
Relative ion intensities (intensity of each ion with respect to the sum of all ions) measured from a 20 mM NH_2_Cl solution as a function of proton transfer reaction time in headspace mode. Standard deviations are shown as error bars (*n* = 3).

The time between the end of headspace introduction and detection (referred to as “reaction time” in Figure S1, which displays the analytical sequence used with the MI FT-ICR mass spectrometer in the present study) was increased from 160 to 2660 ms. Figure 6 shows that NH_3_^35^Cl^+^ and NH_3_^37^Cl^+^ intensities increased when increasing this time from 160 to 560 ms, before stabilizing thereafter; this means that protonation of monochloramine continues inside the ICR cell and quickly reaches a steady state after sample introduction. NH_4_^+^ intensity increases linearly over the whole-time range. This observation, associated with the slow decrease in NH_4_^+^ intensity after removal of the NH_2_Cl solution in the headspace, indicates that adsorbed monochloramine undergoes surface-catalyzed decomposition leading to ammonia.

**Figure 7:**
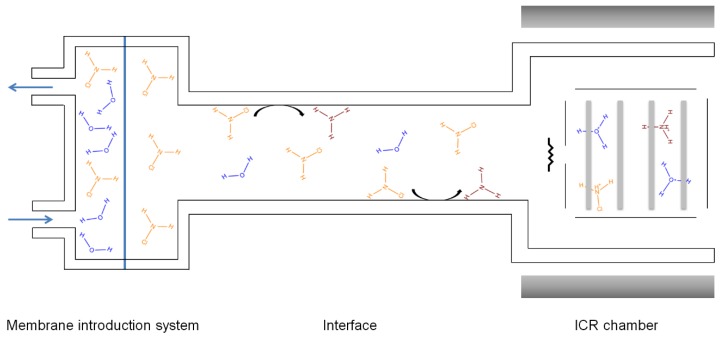
Schematic representation of the adsorption/desorption and surface catalyzed decomposition phenomena.

Detection of ammonium ions is likely due to adsorption/desorption and surface catalyzed decomposition. After pervaporation of monochloramine through the membrane, some NH_2_Cl molecules are adsorbed on the interface walls between the membrane and the ICR cell. A surface catalyzed decomposition of monochloramine adsorbed into ammonia occurs. Then, desorbed NH_3_ molecules are transferred to the ICR cell, where they are protonated into NH_4_^+^ before being detected. The overall process is represented in Figure 7.

## 4. Conclusions

Membrane-introduction FT-ICR mass spectrometry is a potent tool which would allow online monitoring and unequivocal determination of several volatile organic compounds thanks to its high resolution. Nevertheless, the device used in the present study appeared unsuitable for monochloramine analysis, since NH_2_Cl degrades during MIMS analysis. Although decomposition of monochloramine inside the membrane cannot be ruled out, adsorption of NH_2_Cl onto source walls followed by surface-catalyzed decomposition seems more likely to be a predominant phenomenon. The main solutions to minimize adsorption and thus analyte degradation in this type of MIMS system consist in reducing the distance between the membrane inlet system and the ion source or in decreasing the permeate pressure.

## Supplementary Materials

The following are available online at https://www.mdpi.com/1424-8220/20/1/247/s1, Figure S1: Optimized sequence for membrane introduction proton transfer mass spectrometry, Figure S2: Inventory of NH_2_Cl mass spectra obtained by MIMS reported in the literature (A: from Shang and Blatchley (1999) [12]; B: from Riter et al. (2001) [29]; C: from Pope (2006) [30]; D: from Gazda et al. (1993) [31]; E: from Allard et al., 2018 [15]), Figure S3: UV-Vis absorption spectra of 20 mM chlorinated NH_4_Cl solutions at Cl/N = 0.35 (diluted 12.5 times—blue line), Cl/N = 0.70 (diluted 25 times—red line), Cl/N = 1.05 (diluted 37.5 times—green line) and Cl/N = 1.75 (diluted 62.5 times—black line)—Maximum wavelength at 245 nm, Figure S4: UV-Vis absorption spectra of 0.3 mM NH_2_Cl (in blue—maximum wavelength at 245 nm), 0.2 mM NHCl_2_ (in red—maximum wavelength at 295 nm) and 0.2 mM NCl_3_ (in green—maximum wavelength at 340 nm), Figure S5: Mass spectrum of a mixture of 20 mM NH_4_Cl and 35 mM HOCl—Isotopic distribution of dichloramine and fragmented trichloramine, Table S1: Literature backgrounds regarding organic chloramines analysis using the MIMS method, Table S2: Theoretical mass, calculated mass and mass error for ions detected during chlorination of a 20 mM NH_4_Cl solution.
