# Supplementary material for: Tracking Monochloramine Decomposition in MIMS Analysis
Source: Sensors (Basel). 2019 Dec 31;20(1):247. doi: 10.3390/s20010247 (PMC6982842; doi:10.3390/s20010247)
Supplement: Supplementary file 1 [file sensors-20-00247-s001.pdf]

Supplementary Information

Tracking Monochloramine Decomposition in MIMS Analysis

Adrien Roumiguières [1,2], Said Kinani [1] and Stéphane Bouchonnet [2,*]

[1] Laboratoire National d'Hydraulique et Environnement (LNHE), Division Recherche et Développement, Electricité de France (EDF), 6 Quai Watier, 78401 Chatou CEDEX 01, France; adrien.roumiguieres@polytechnique.edu (A.R.); said.kinani@edf.fr (S.K.)

[2] Laboratoire de Chimie Moléculaire, CNRS, Institut polytechnique de Paris, Route de Saclay, 91128 Palaiseau, France

* Correspondence: stephane.bouchonnet@polytechnique.edu; Tel.: +33-(0)1-69-33-48-05

Received: 28 October 2019; Accepted: 26 December 2019; Published: date

Contents of the Supporting Information

Figure S1. Optimized sequence for membrane introduction proton transfer mass spectrometry.

Figure S2. Inventory of NH_2Cl mass spectra obtained by MIMS reported in the literature (A: from Shang and Blatchley (1999); B: from Riter et al. (2001); C: from Pope (2006); D: from Gatda et al. (1993); E: from Allard et al., 2018).

Figure S3. UV-Vis absorption spectra of 20 mM chlorinated NH_4Cl solutions at Cl/N = 0.35 (diluted 12.5 times – blue line), Cl/N = 0.70 (diluted 25 times – red line), Cl/N = 1.05 (diluted 37.5 times – green line) and Cl/N = 1.75 (diluted 62.5 times - black line) - Maximum wavelength at 245 nm.

Figure S4. UV-Vis absorption spectra of 0.3 mM NH_2Cl (in blue - maximum wavelength at 245 nm), 0.2 mM $NHCl_2$ (in red - maximum wavelength at 295 nm) and 0.2 mM NCl_3 (in green - maximum wavelength at 340 nm).

Figure S5. Mass spectrum of a mixture of 20 mM NH_4Cl and 35 mM HOCl - Isotopic distribution of dichloramine and fragmented trichloramine.

Table S1. Literature backgrounds regarding organic chloramines analysis using the MIMS method

Table S2. Theoretical mass, calculated mass and mass error for ions detected during chlorination of a 20 mM NH_4Cl solution.

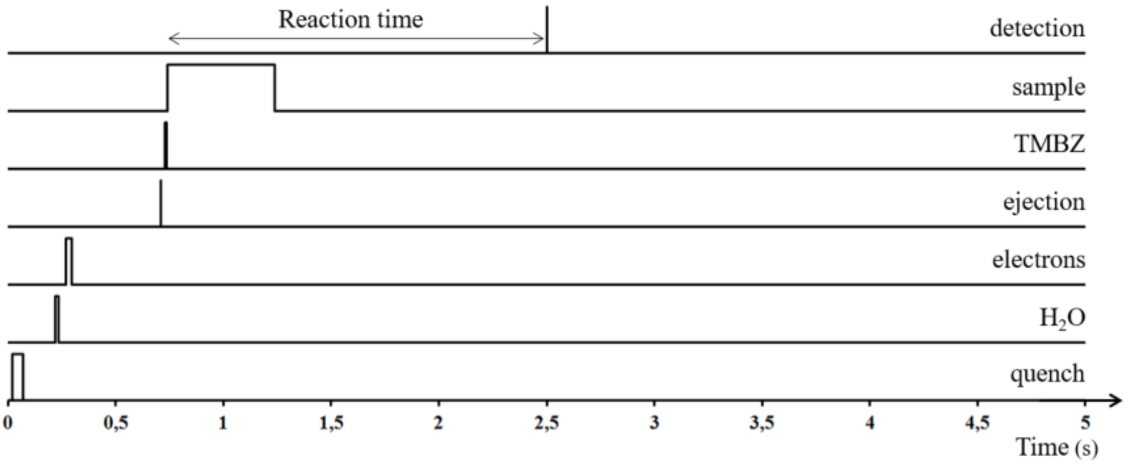

Figure S1. Optimized sequence for membrane introduction proton transfer mass spectrometry.

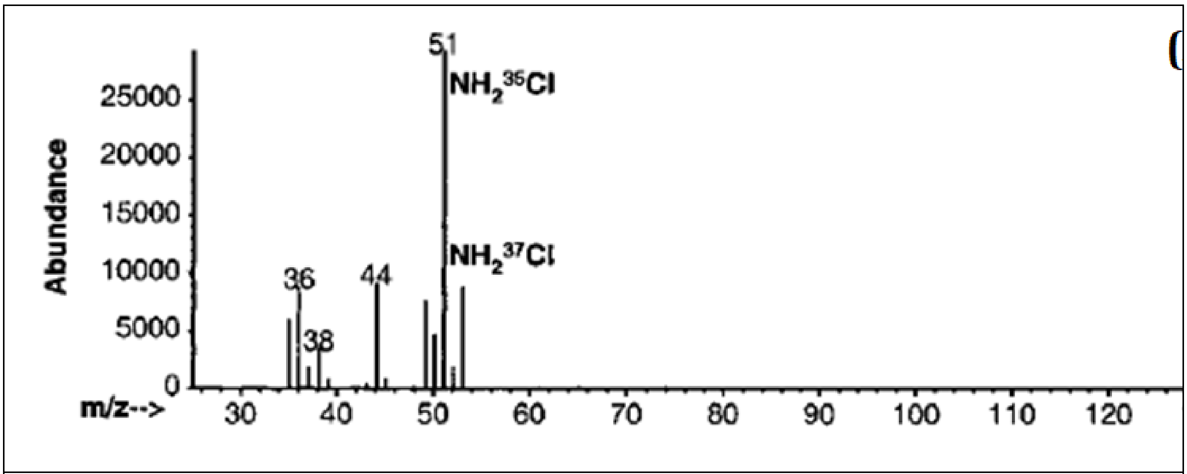
51
$NH_2{}^{35}Cl$
$NH_2{}^{37}Cl$
Abundance
25000
20000
15000
10000
5000
0
36
38
44
m/z-->
30
40
50
60
70
80
90
100
110
120

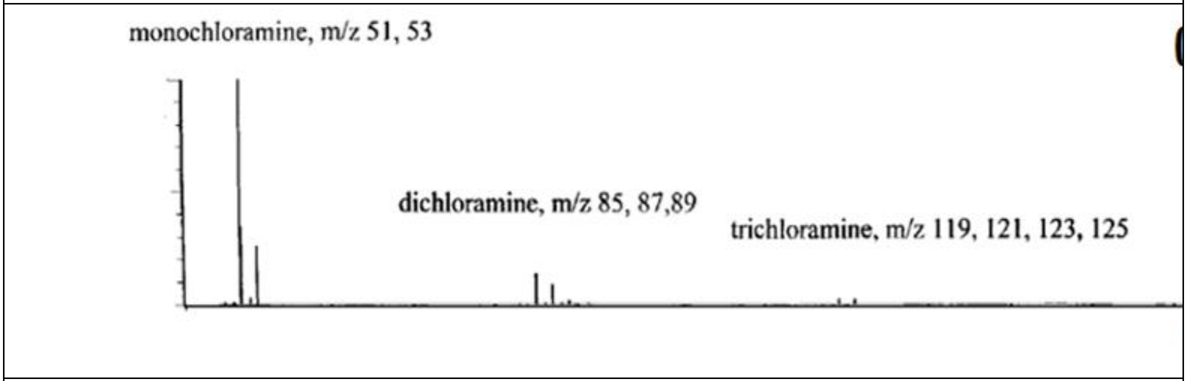
monochloramine, m/z 51, 53
dichloramine, m/z 85, 87,89
trichloramine, m/z 119, 121, 123, 125

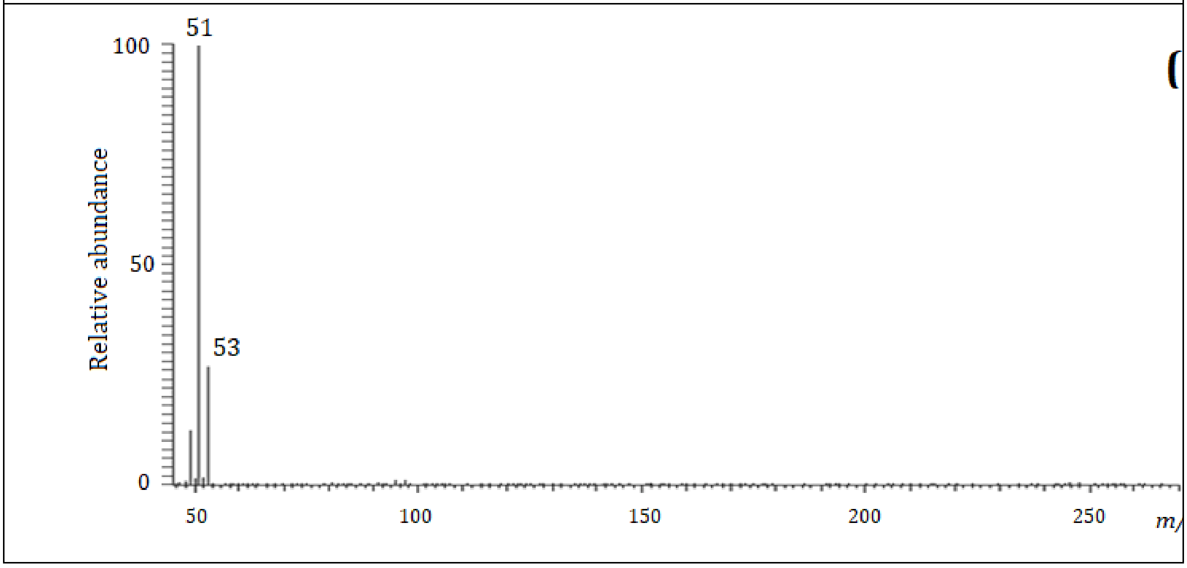
51
100
50
0
Relative abundance
53
50
100
150
200
250

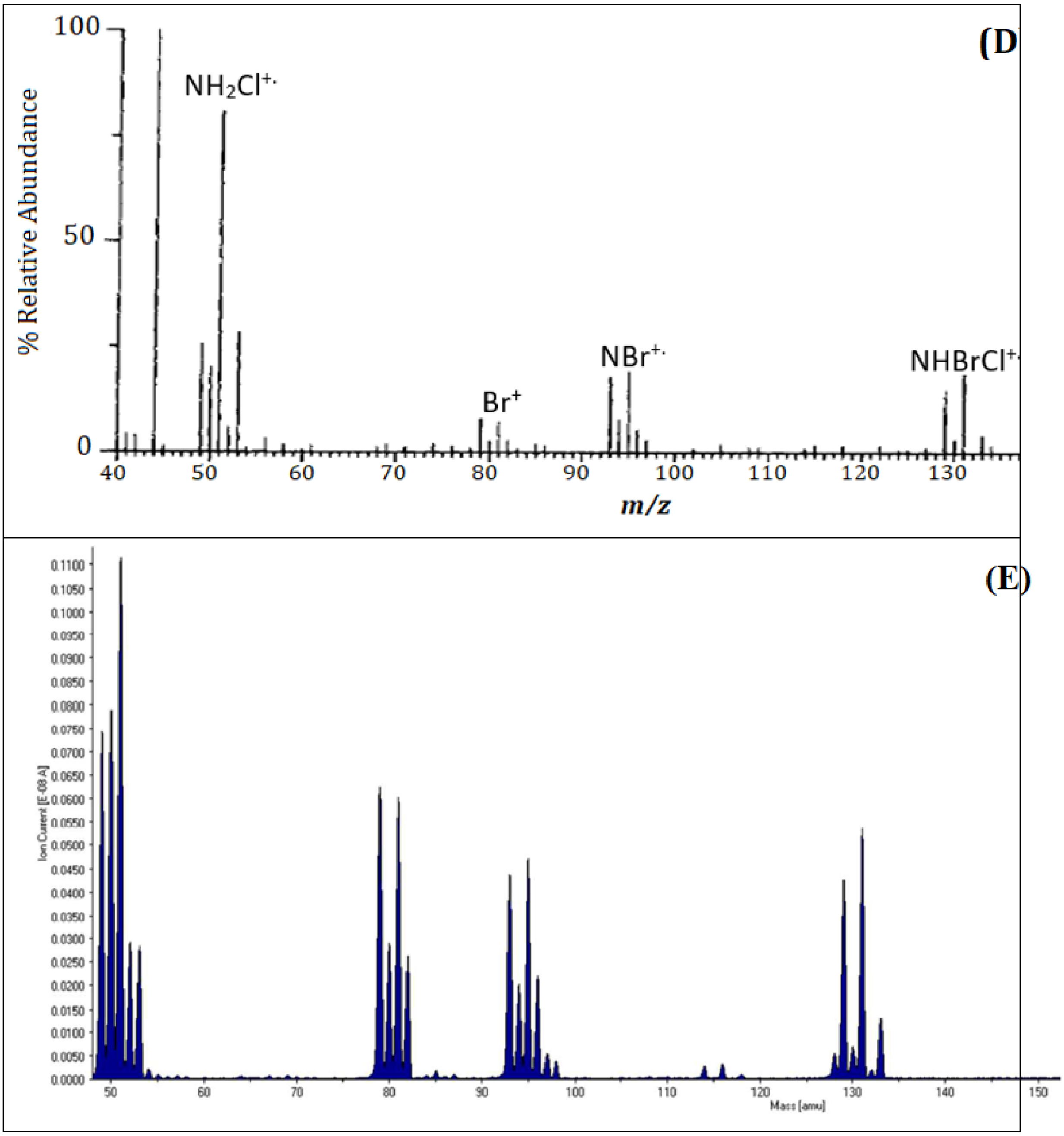

Figure S2. Inventory of NH_2Cl mass spectra obtained by MIMS reported in the literature (A: from Shang and Blatchley (1999); B: from Riter et al. (2001); C: from Pope (2006); D: from Gatda et al. (1993); E: from Allard et al., 2018).

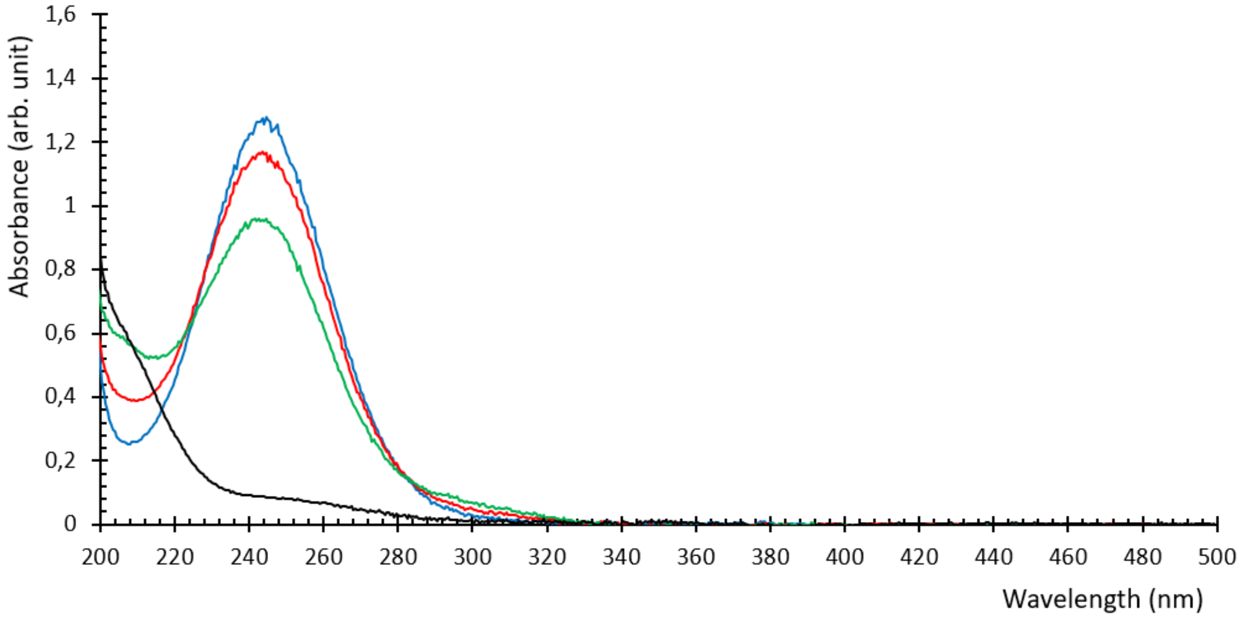

Figure S3. UV-Vis absorption spectra of 20 mM chlorinated NH_4Cl solutions at Cl/N = 0.35 (diluted 12.5 times – blue line), Cl/N = 0.70 (diluted 25 times – red line), Cl/N = 1.05 (diluted 37.5 times – green line) and Cl/N = 1.75 (diluted 62.5 times - black line) - Maximum wavelength at 245 nm.

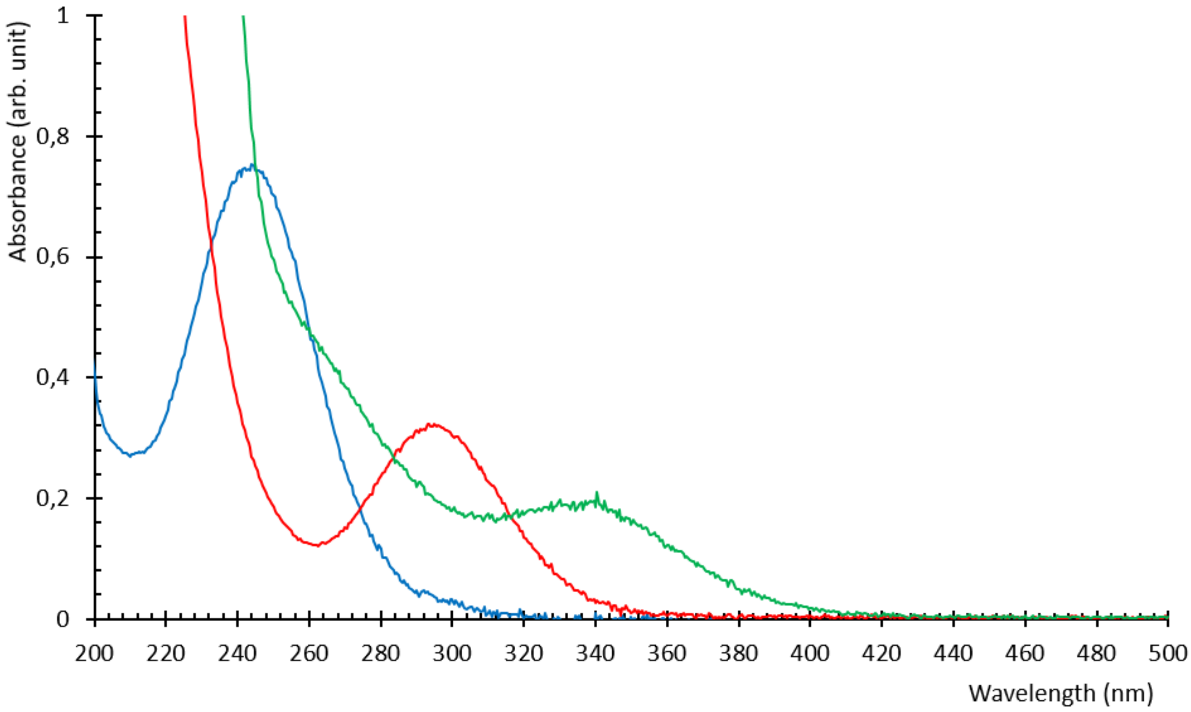

Figure S4. UV-Vis absorption spectra of 0.3 mM NH_2Cl (in blue - maximum wavelength at 245 nm), 0.2 mM $NHCl_2$ (in red - maximum wavelength at 295 nm) and 0.2 mM NCl_3 (in green - maximum wavelength at 340 nm).

.

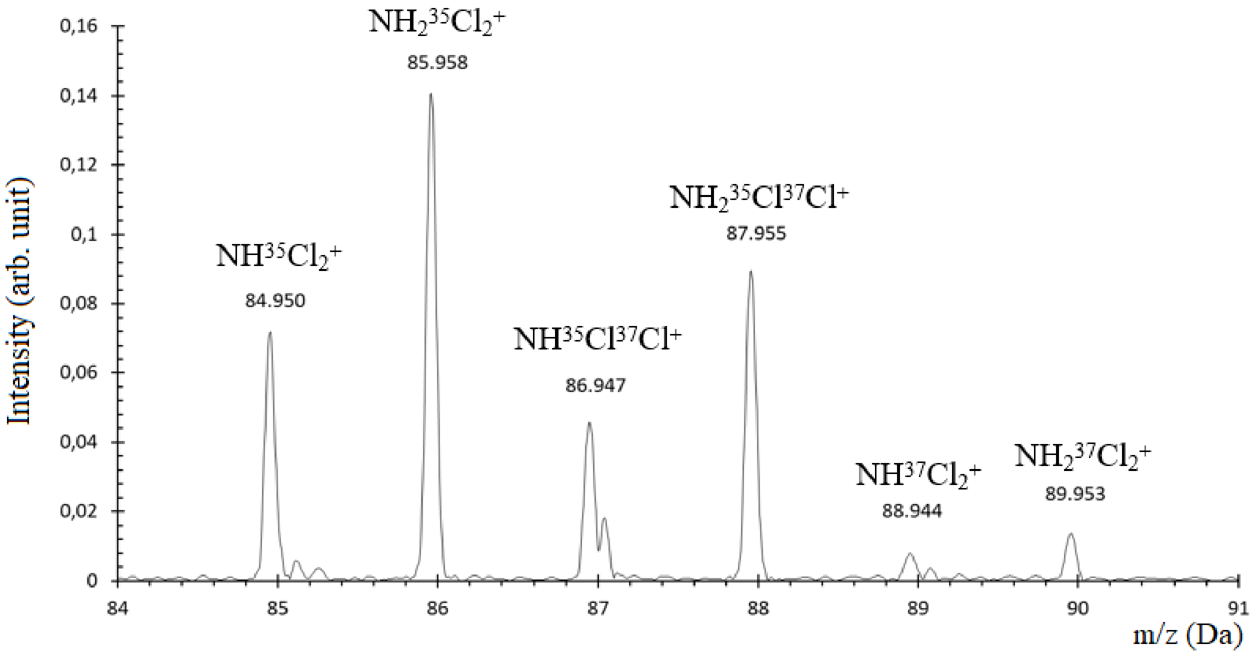

Figure S5. Mass spectrum of a mixture of 20 mM NH_4Cl and 35 mM HOCl - Isotopic distribution of dichloramine and fragmented trichloramine.

Table S1. Literature backgrounds regarding monochloramine analysis using the MIMS method.

Analyte	Membrane	MS analyzer	Ionization mode	References
NH_2Cl	PDMS sheet (Dow Corning, 0.12 mm)	t-quad	EI (35–300 m/z)	Gazda et al. (1993)
NH_2Cl	PDMS hollow fiber (Baxter, 0.25 × 0.47 × 60 mm)	s-quad	EI (35–125 m/z)	Shang & Blatchley (1999)
NH_2Cl	PDMS hollow fiber (Dow Corning, 0.64 × 1.19 × 35 mm)	ion trap	EI (40–170 m/z)	Riter et al. (2001)
NH_2Cl	PDMS hollow fiber (Baxter, 0.25 × 0.47 × 60 mm)	s-quad	EI (35–125 m/z)	Yang & Shang (2004)
NH_2Cl	PDMS hollow fiber (Dow Corning, 0.64 × 1.19 × 60 mm)	ion trap	EI (45–270 m/z)	Pope (2006)
NH_2Cl	PDMS hollow fiber (Baxter, 0.64 × 1.19 × 60 mm)	s-quad	EI (35–125 m/z)	Lee et al. (2007)
NH_2Cl	PDMS hollow fiber (Baxter, 0.25 × 0.47 × 60 mm)	s-quad	EI (49–200 m/z)	Li & Blatchley (2007)
NH_2Cl	Information not supplied	s-quad	EI (49–200 m/z)	Weaver et al. (2009)
NH_2Cl	PDMS sheet (Goodfellow, 0.125 × 1.5 × 40 mm)	FT-ICR	CI (8–300 m/z)	Louarn et al. (2018)
NH_2Br	PDMS hollow fiber (Dow Corning, 0.64 × 1.19 × 60 mm)	ion trap	EI (45–270 m/z)	Pope (2006)
NH_2Cl	Silicon membrane (dimensions not supplied)	s-quad	EI SIM (m/z 53)	Allard et al. (2018)

Abbreviations: PDMS: Poly(dimethylsiloxane), SIM: Single-ion monitoring, EI: Electron ionization, CI: Chemical ionization, s-quad: Single quadrupole mass spectrometer.

Table S2. Theoretical mass, calculated mass and mass error for ions detected during chlorination of 20 mM NH_4Cl solution.

Ions	Theoretical mass (Da)	Calculated mass (Da)	Mass error (Da)
NH_4^+	18.034	18.037	0.003
$NH_3{}^{35}Cl^+$	51.995	51.998	0.003
$NH_3{}^{37}Cl^+$	53.992	53.995	0.003
$NH^{35}Cl_2{}^+$	84.949	84.950	0.001
$NH_2{}^{35}Cl_2{}^+$	85.956	85.958	0.002
$NH^{35}Cl^{37}Cl^+$	86.946	86.947	0.001
$NH_2{}^{35}Cl^{37}Cl^+$	87.954	87.955	0.001
$NH^{37}Cl_2{}^+$	88.943	88.944	0.001
$NH_2{}^{37}Cl_2{}^+$	89.951	89.953	0.002

References

Allard, S.; Hu, W.; Le Menn, J.-B.; Cadee, K.; Gallard, H.; Croué, J.-P. Method development for quantification of bromochloramine using membrane introduction mass spectrometry. *Environ. Sci. Technol.* **2018**, *52*, 7805–7812.

Gazda, M.; Dejarme, L.E.; Choudhury, T.K.; Cooks, R.G.; Mergerum, D.W. Mass spectrometric evidence for the formation of bromochloramine and N-bromo-N-chlormethylamine in aqueous solution. *Environ. Sci. Technol.* **1993**, *27*, 557–561.

Lee, W.; Westerhoff, P.; Yang, X.; Shang, C. Comparison of colorimetric and membrane introduction mass spectrometry techniques for chloramine analysis. *Water Res.* **2007**, *41*, 3097–3102.

Li, J.; Blatchley, E.R. Volatile disinfection byproduct formation resulting from chlorination of organic-nitrogen precursors in swimming pools. *Environ. Sci. Technol.* **2007**, *41*, 6732–6739.

Louarn, E.; Asri-Idlibi, A.M.; Leprovost, J.; Héninger, M.; Mestdagh, H. Evidence of reactivity in the membrane for the unstable monochloramine during MIMS analysis. *Sensors* **2018**, *18*, 4252.

Pope, P.G. Haloacetic acid formation during chloramination: role of environmental conditions, kinetics, and haloamine chemistry, Ph.D. Thesis, The University of Texas, Austin, Texas, 2006.

Riter, L.S.; Charles, L.; Turowski, M.; Cooks, R.G. External interface for trap-and release membrane introduction mass spectrometry applied to the detection of inorganic chloramines and chlorobenzenes in water. *Rapid Commun. Mass Spectrom.* **2001**, *15*, 2290–2295.

Shang, C.; Blatchley, E.R. Differentiation and quantification of free chlorine and inorganic chloramines in aqueous solution by MIMS. *Environ. Sci. Technol.* **1999**, *33*, 2218–2223.

Weaver, W.A.; Li, J.; Wen, Y.; Johnston, J.; Blatchley, M.R.; Blatchley, E.R. Volatile disinfection by-products analysis from chlorinated indoor swimming pools. *Water Res.* **2009**, *43*, 3308–3318.

Yang, X.; Shang, C. Chlorination byproduct formation in the presence of humic acid, model nitrogenous organic compounds, ammonia, and bromide. *Environ. Sci. Technol.* **2004**, *38*, 4995–5001.
